# Supplementary material for: Confirming the Molecular Basis of the Solvent Extraction of Cadmium(II) Using 2-Pyridyl Oximes through a Synthetic Inorganic Chemistry Approach and a Proposal for More Efficient Extractants
Source: Molecules. 2022 Feb 28;27(5):1619. doi: 10.3390/molecules27051619 (PMC8911866; doi:10.3390/molecules27051619)
Supplement: Supplementary file 1 [file molecules-27-01619-s001.zip › molecules-1605324-supplementary.pdf]

Supplementary materials to

Confirming the Molecular Basis of the Solvent Extraction of Cadmium(II) using 2-pyridyl Oximes through a Synthetic Inorganic Chemistry Approach and a Proposal for more Efficient Extractants

Anastasia Routzomani [1], Zoi G. Lada [1,2], Varvara Angelidou [1], Catherine P. Raptopoulou [3], Vassilis Psycharis [3,*], Konstantis F. Konidaris [4,*], Christos T. Chasapis [2,5,*] and Spyros P. Perlepes [1,2,*]

[1] Department of Chemistry, University of Patras, 265 04 Patras, Greece; a.routzomani@gmail.com (A.R.); varvara_angelidou@hotmail.com (V.A.)

[2] Institute of Chemical Engineering Sciences (ICE-HT), Foundation for Research and Technology-Hellas, (FORTH), Platani, P.O. Box 1414, 265 04 Patras, Greece; zoilada@iceht.forth.gr (Z.G.L.)

[3] Institute of Nanoscience and Nanotechnology, NCSR "Demokritos", 153 10 Aghia Paraskevi Attikis, Greece; c.raptopoulou@inn.demokritos.gr

[4] Department of Science and High Technology and INSTM, University of Insubria, 22 100 Como, Italy

[5] NMR Facility, Instrumental Analysis Laboratory, School of Natural Sciences, University of Patras, 265 04 Patras, Greece

* Correspondence: v.psycharis@inn.demokritos.gr (V.P.); konstantis.konidaris@uninsubria.it (K.F.K); cchasapis@upatras.gr (C.T.C.); perlepes@patreas.upatras.gr (S.P.P.); Tel.: +30 2610996730 (S.P.P.); +30 2610 996261 (C.T.C.); +39 0312386472 (K.F.K.); +30 2106503346 (V.P.)

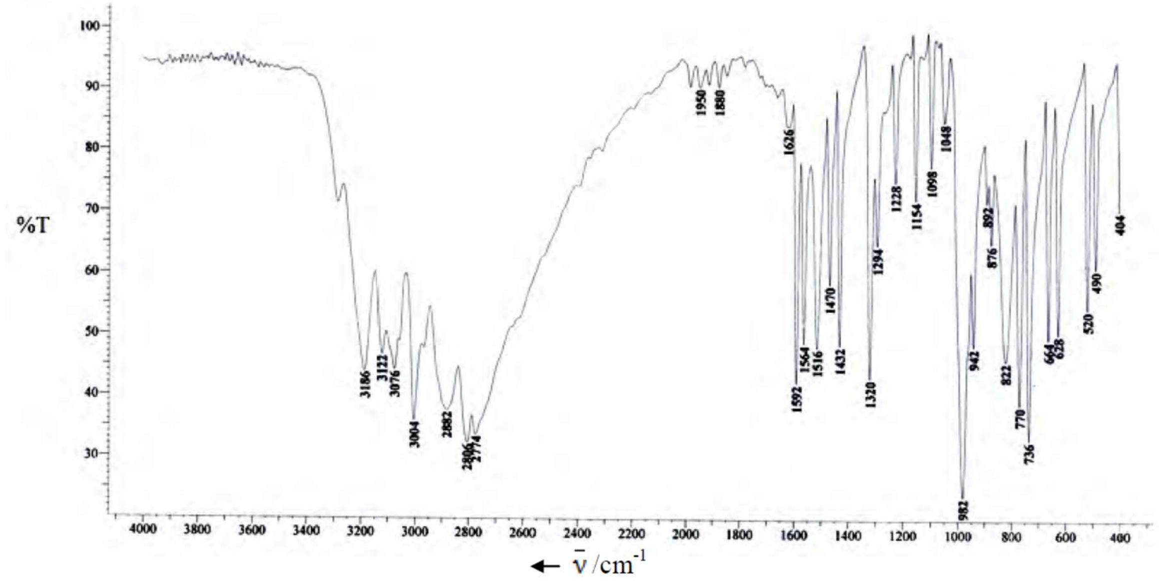

Figure S1. The FT-IR spectrum (KBr/cm^{-1}) of free paoH.

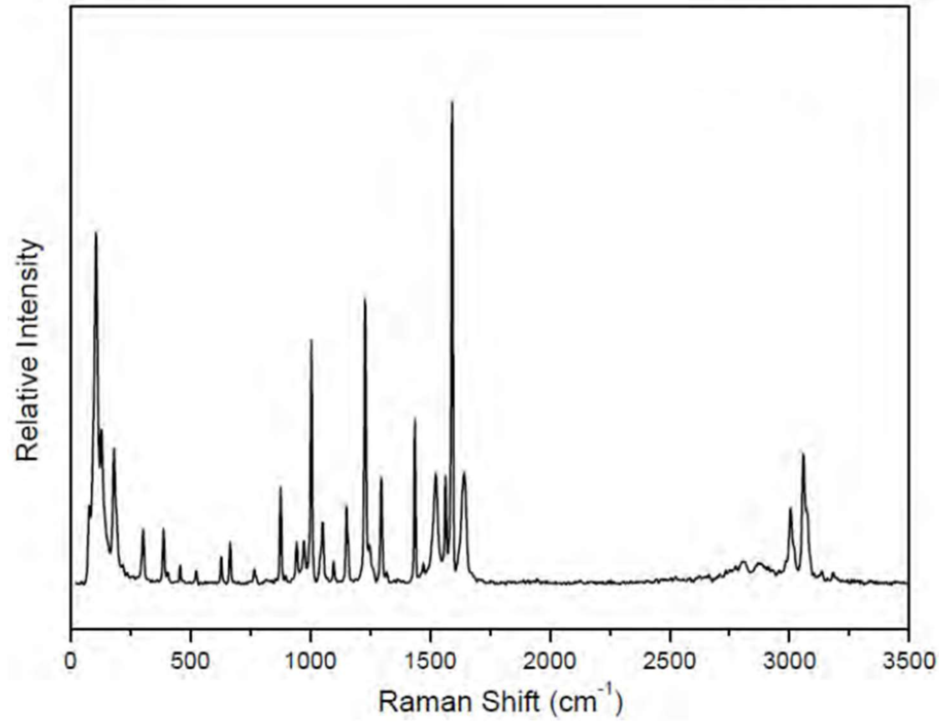

Figure S2. The FT-Raman spectrum of free paoH.

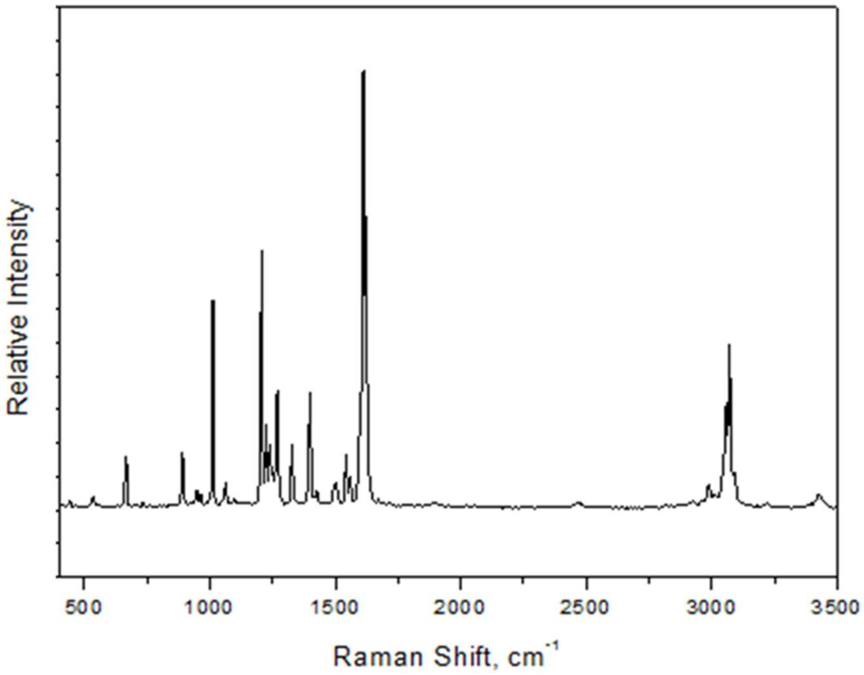

Figure S3. The FT-Raman spectrum of $\{[CdI_2(4paoH)_2]\}_n$ (**3**).

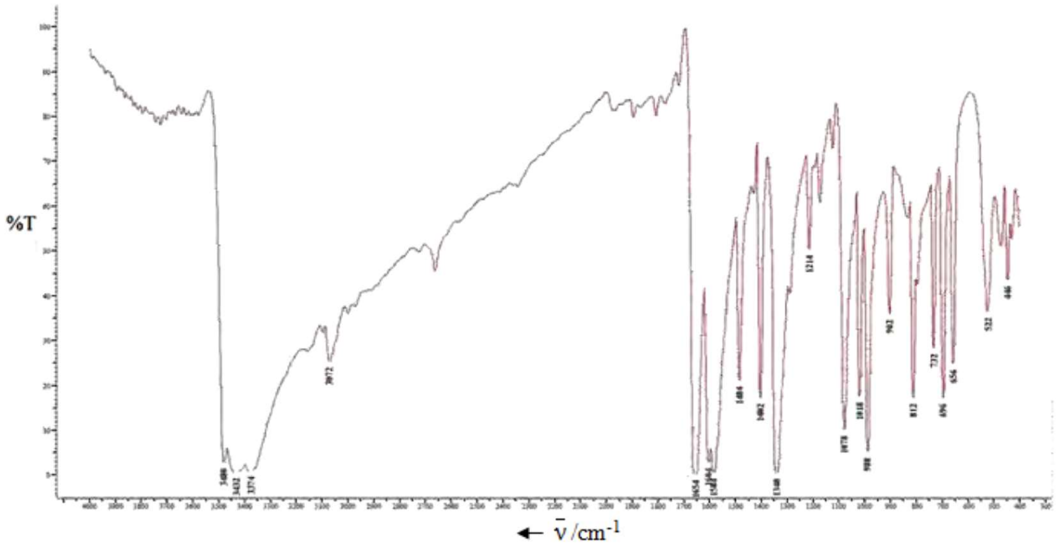

Figure S4. The FT-IR spectrum (KBr/cm^{-1}) of compound $[CdI_2(LH_4)]$ (**5a**).

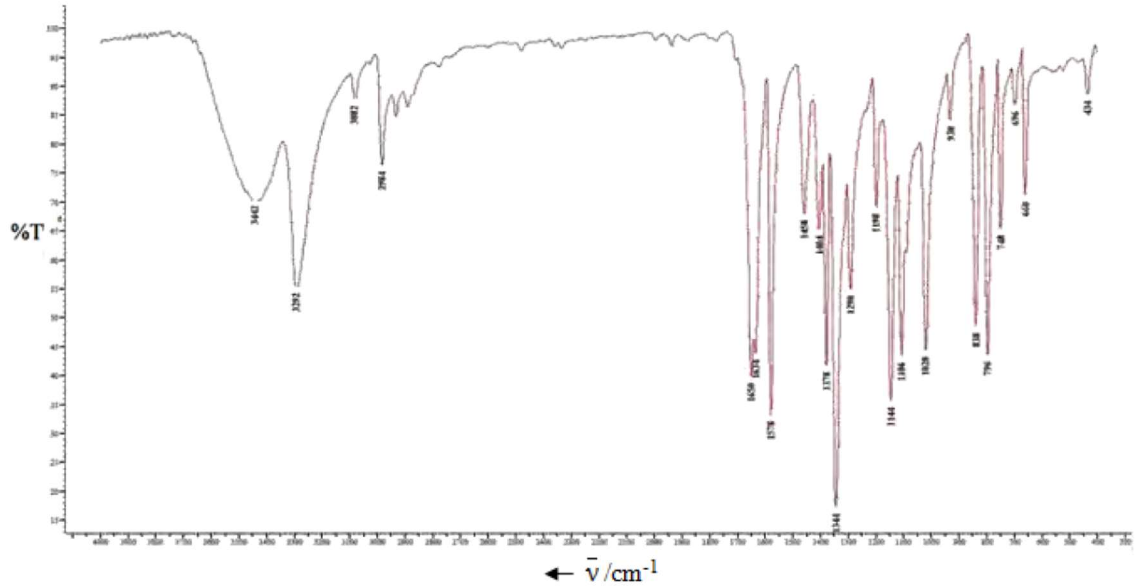

Figure S5. The FT-IR spectrum (KBr/cm^{-1}) of complex $[CdI_2(L'H)_2]$ (**5**).

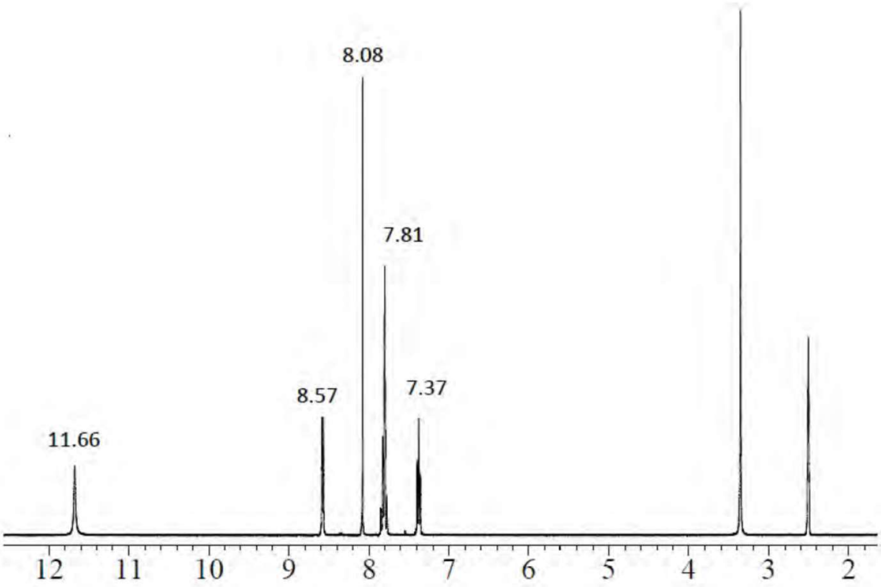

Figure S6. The ^1H NMR spectrum (δ/ppm) of the free ligand 2paoH in d_6-DMSO.

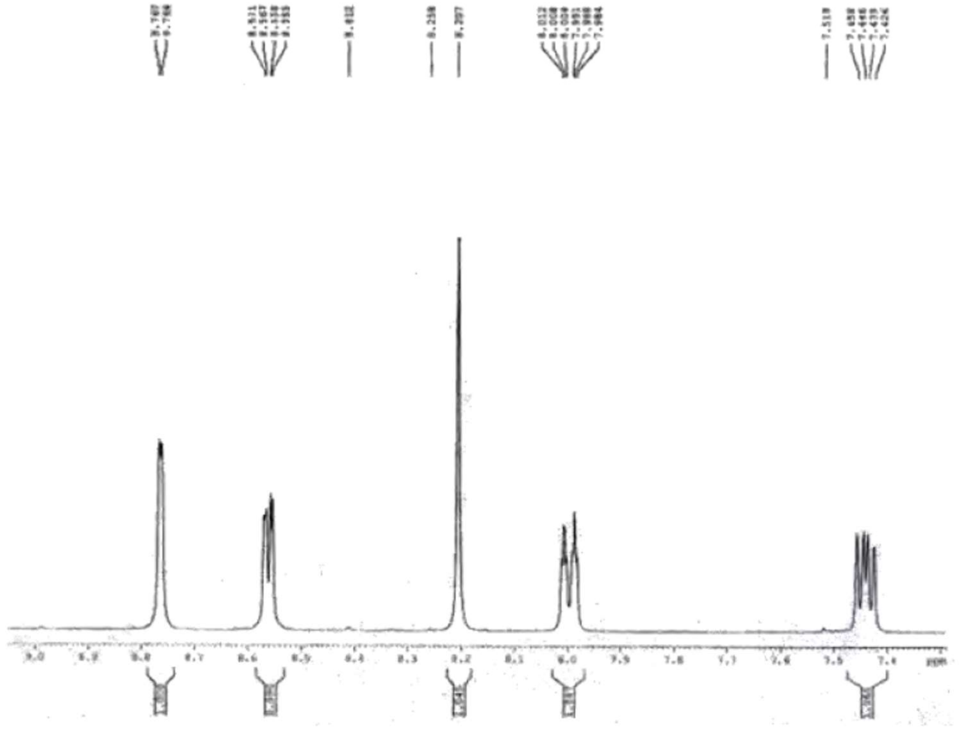

Figure S7. The ^{1}H NMR spectrum (δ/ppm) of complex $\{[CdI_2(3paoH)]\}_n$ (**2**)$_x$ in d_6-DMSO in the 9.0-7.33 ppm region.

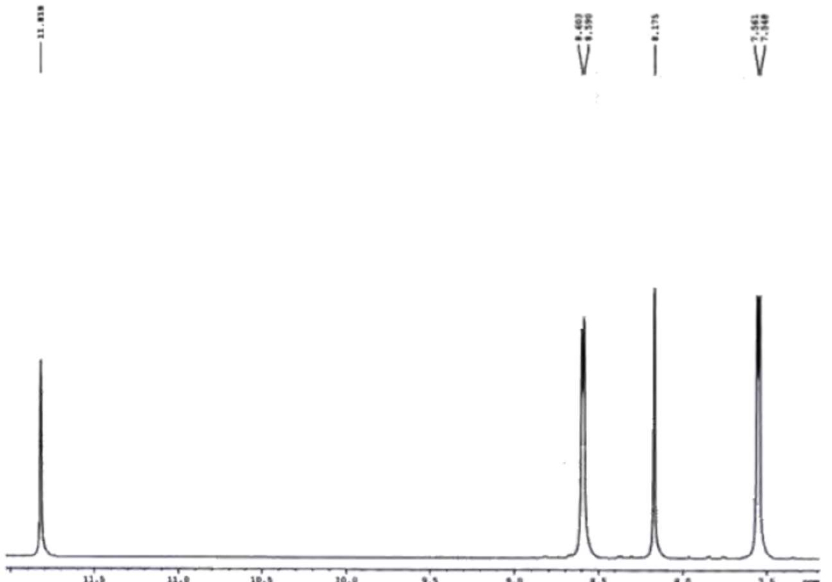

Figure S8. The ^{1}H NMR spectrum (δ/ppm) of compound $\{[CdI_2(4paoH)_2]\}_n$ (**3**) in d_6-DMSO in the 12.0-7.3 ppm region.

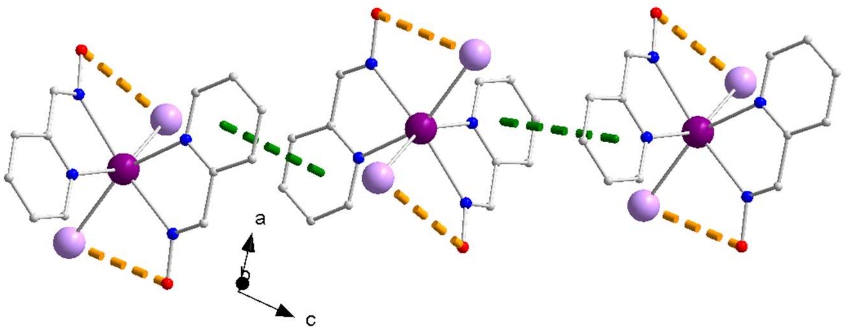

Figure S9. A portion of one chain, parallel to the crystallographic direction [101], in the crystal structure of [$CdI_2(2paoH)_2$] (**1**). The thick dashed orange and green lines represent the intramolecular O1-H(O1)···I1′ and O1′-H(O1′)…I1 hydrogen bonds [(′)= -*x*, *y*, -*z*+1/2] and the π-π interactions, respectively.

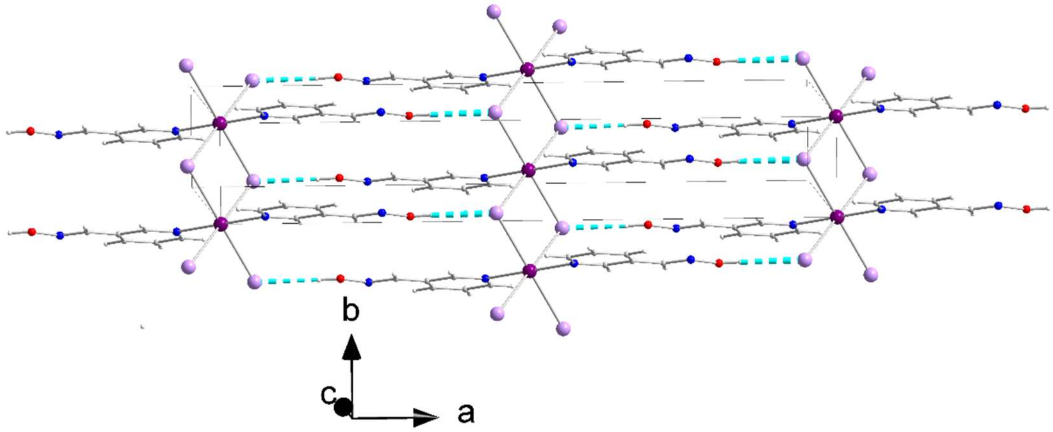

Figure S10. Layers of chains parallel to the (110) plane in the crystal structure of {[$CdI_2(3paoH)_2$]}$_n$ (**2**). The layers are formed through O-H···I hydrogen bonds (thick dashed cyan lines); see text for more details.

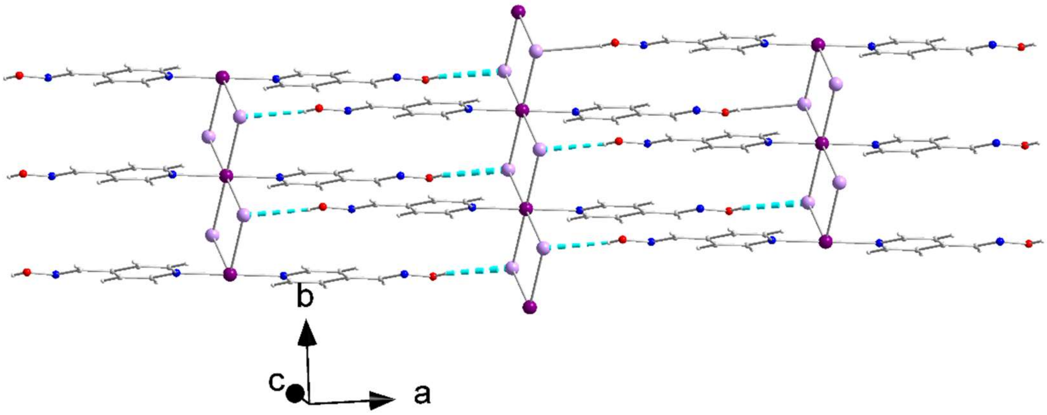

Figure S11. Layers of chains parallel to the (110) plane in the crystal structure of {[$CdI_2(4paoH)_2$]}$_n$ (**3**). The layers are formed through O-H···I hydrogen bonds (thick dashed cyan lines); see text for the metric parameters.

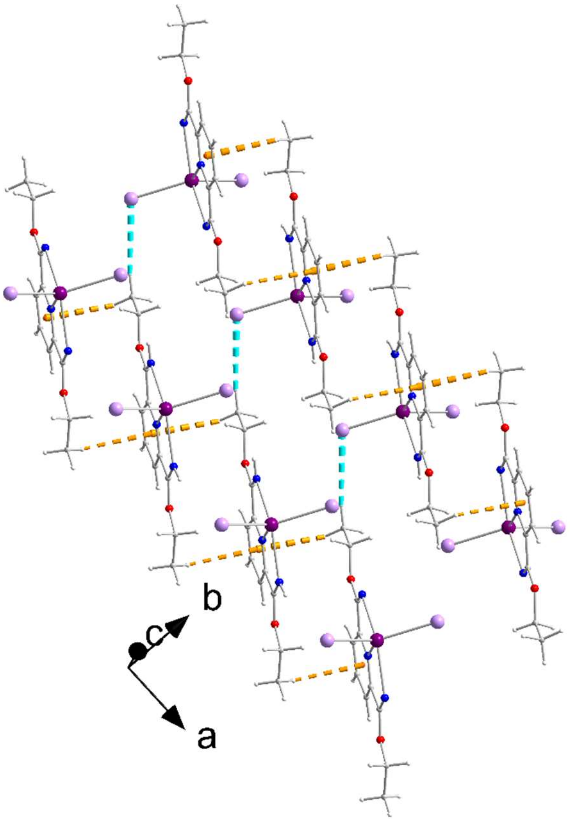

Figure S12. Formation of chains parallel to the *a* axis and layers parallel to the (001) crystallographic plane in the crystal structure of complex [$CdI_2(L'H_2)$] (**5**). The dashed thick orange line and the cyan lines represent the C(methyl)-H···π and C(methyl)-H···I interactions; see Table S2 for metric parameters.

Table S1. Hydrogen bonding interactions (Å, deg) in the crystal structure of [$CdI_2(dapdoH_2)$]·2EtOH (**4**·2EtOH).[a]

D—H···A	d(D···A)	d(D···H)	d(H···A)	<DHA	Symmetry Code of A
O1—H(O1)···O4	2.614 (4)	0.84	1.79	168	*x,y,z*
O2—H(O2)···O3	2.622 (3)	0.84	1.82	158	*x,y,z*
C4—H(C4)···I2	3.889 (3)	0.95	3.08	144	-*x*, -*y*+2, -*z*+2
C9—H_C(C9)···O2	3.440 (4)	0.98	2.54	152	-*x*+1, -*y*+1, -*z*+2

[a] For more details, see text. D= donor; A= acceptor.

Table S2. Hydrogen bonding interactions (Å, deg) in the crystal structure of [$CdI_2(L'H_2)$] (**5**).

D—H···A	d(D···A)	d(D···H)	d(H···A)	<DHA	Symmetry Code of A
C1—H_A(C1)···I1	4.143(6)	0.98	3.17	171	-*x*+1, -*y*+1, -*z*+1
C1—H_C(C1)···Cg1[a]	3.721(6)	0.98	2.95	136	*x*+1, *y*, *z*

[a] C_g1 is the centroid of the ring N2C4C5C6C5'C4' [(')= -*x*, -*y*+3/2, *z*]. D= donor; A= acceptor.
